# Cytoplasmic sphingosine-1-phosphate pathway modulates neuronal autophagy

**DOI:** 10.1038/srep15213

**Published:** 2015-10-19

**Authors:** Jose Felix Moruno Manchon, Ndidi-Ese Uzor, Yuri Dabaghian, Erin E. Furr-Stimming, Steven Finkbeiner, Andrey S. Tsvetkov

**Affiliations:** 1 Department of Neurobiology and Anatomy, University of Texas Medical School, Houston, TX 77030; 2 The Jan and Dan Duncan Neurological Research Institute, Baylor College of Medicine, Houston, TX 77030; 3 Department of Computational and Applied Mathematics, Rice University, Houston, TX 77005; 4 Department of Neurology, University of Texas Medical School, Houston, TX 77030; 5 Gladstone Institute of Neurological Disease and the Taube/Koret Center for Neurodegenerative Disease Research, San Francisco, CA 94158; 6 Departments of Neurology and Physiology, University of California, San Francisco, CA 94143; 7 The University of Texas Graduate School of Biomedical Sciences, Houston, TX 77030.

## Abstract

Autophagy is an important homeostatic mechanism that eliminates long-lived proteins, protein aggregates and damaged organelles. Its dysregulation is involved in many neurodegenerative disorders. Autophagy is therefore a promising target for blunting neurodegeneration. We searched for novel autophagic pathways in primary neurons and identified the cytosolic sphingosine-1-phosphate (S1P) pathway as a regulator of neuronal autophagy. S1P, a bioactive lipid generated by sphingosine kinase 1 (SK1) in the cytoplasm, is implicated in cell survival. We found that SK1 enhances flux through autophagy and that S1P-metabolizing enzymes decrease this flux. When autophagy is stimulated, SK1 relocalizes to endosomes/autophagosomes in neurons. Expression of a dominant-negative form of SK1 inhibits autophagosome synthesis. In a neuron model of Huntington’s disease, pharmacologically inhibiting S1P-lyase protected neurons from mutant huntingtin-induced neurotoxicity. These results identify the S1P pathway as a novel regulator of neuronal autophagy and provide a new target for developing therapies for neurodegenerative disorders.

Macroautophagy (hereafter referred to as autophagy) is an intracellular turnover pathway that is used to remove long-lived and aggregated proteins, organelles, and parasites from the cell^1^. With this pathway, damaged or senescent cytoplasmic contents are sequestered in double-membrane vesicles called autophagosomes. Biogenesis and maturation of autophagosomes begins with the phagophore, an autophagosome precursor^2^, whose membrane lipids can be derived from multiple sources, including the endoplasmic reticulum (ER)^3^, clathrin-coated vesicles from the plasma membrane^4^, the outer membrane of mitochondria^5^, and the trans-Golgi network^6^. The autophagosome then fuses with early or late endosomes or with lysosomes for degradation of sequestered contents.

Autophagy plays a critical role in neuronal survival^7^. For example, blocking autophagy in neurons in the mouse central nervous system leads to protein aggregation and neurodegeneration^8^,^9^. Autophagosomes have been observed in many neurodegenerative disorders^10^. Small molecules that stimulate autophagy ameliorate pathogenic changes in a number of neurodegenerative disorders^1^,^11^,^12^. Despite the mounting evidence demonstrating the importance of autophagy in neurons, the molecular players that regulate neuronal autophagy are not fully understood.

In a search for novel regulators of autophagy in neurons, we investigated whether sphingosine kinase 1 (SK1), which regulates autophagy in cancer cells^13^, is necessary for autophagy in neurons. There are two sphingosine kinases in mammalian cells, cytosolic SK1 and nuclear SK2. In non-neuronal cells stimulated with growth factors, SK1 translocates to the plasma membrane where it phosphorylates sphingosine to produce sphingosine-1-phosphate (S1P)^14^. S1P regulates a wide variety of cellular processes, including cell survival^15^.

In the present study, we found that, in neurons, expression of SK1 enhanced the formation of pre-autophagosomal structures and increased flux through the autophagic pathway, and enzymes that metabolize S1P, S1P-phosphatase (S1PP) and S1P-lysase (S1PL), inhibited autophagy. Pharmacological stimulation of autophagy triggered the relocation of SK1 to organelles positive for endosomal or autophagosomal markers or both. The existence of these three organelle pools suggests that endosomes and autophagosomes fuse in neurons undergoing autophagy. Interestingly, expression of dominant-negative SK1 inhibited autophagosome synthesis in neurons, suggesting that SK1 plays a role in the biogenesis of autophagosomes.

In non-neuronal cells, S1P can mobilize Ca^2+^ from the ER^16^,^17^, which in turn may upregulate autophagy^18^,^19^. The ER itself can provide a membrane platform for building an autophagosome^3^,^20^. We hypothesized that endosomes might be the source of S1P that orchestrates the ER-dependent biogenesis of autophagosomes in neurons. We demonstrated that endosomes make contacts with the ER in cells undergoing autophagy, suggesting that endosomes might provide the ER with S1P for the biogenesis of autophagosomes. Remarkably, both S1PP and S1PL are located on the ER, ensuring that unwanted S1P can be locally metabolized to stop S1P signaling.

Finally, we found that SK1 and S1PL regulate the degradation of mutant huntingtin, the protein that causes Huntington’s disease (HD), thus demonstrating a role for the S1P in a relevant and highly penetrant neurodegenerative phenotype. We also demonstrated that pharmacologically inhibiting S1P-lyase protected neurons from mutant huntingtin-induced neurotoxicity. Our results demonstrate a novel autophagic pathway in neurons and identify a new target for developing therapies for neurodegenerative disorders.

## Results

### Expression of SK1 stimulates autophagy in primary neurons

**Figure 1:**
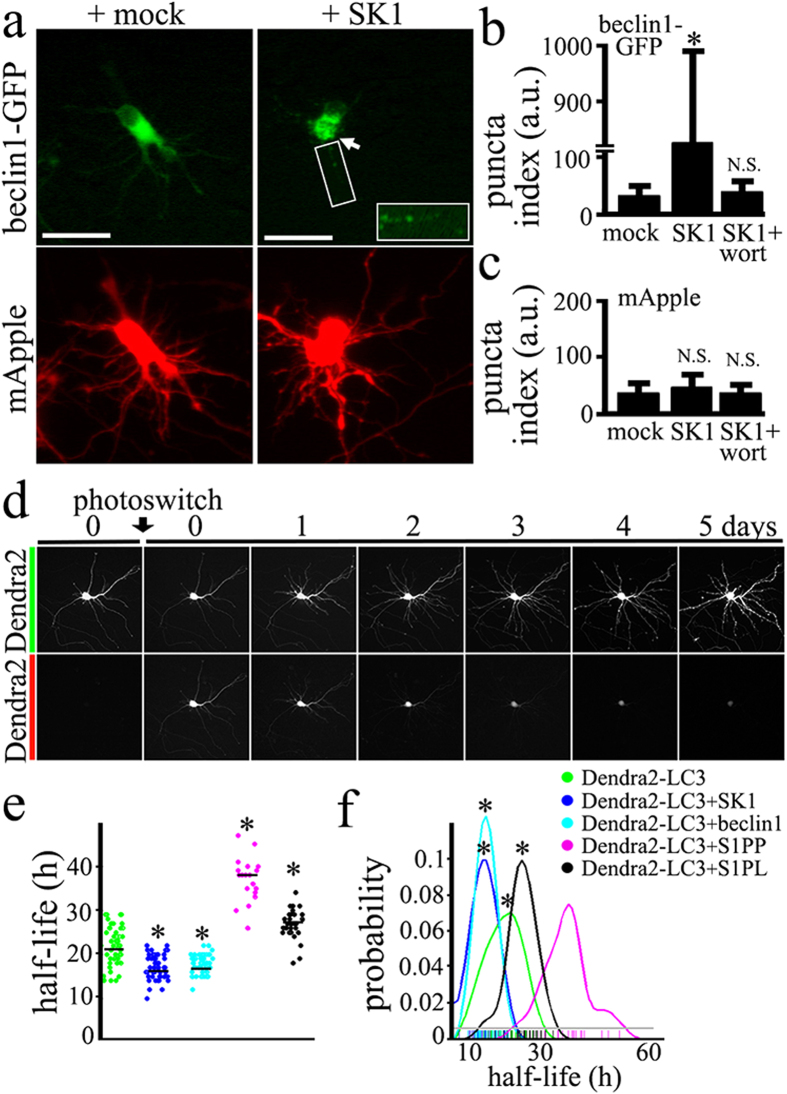
SK1 enhances autophagy in primary neurons. (**a**) Autophagy was effectively induced in primary cortical neurons by overexpression of untagged SK1. Two neuronal cohorts were transfected with a red marker of morphology, mApple (bottom panel), a marker of autophagy, beclin1-GFP (top panel), and cotransfected either with an empty plasmid (mock; left panel) or untagged SK1 (right panel). Live neurons were analyzed with epifluorescence. Note the changes in beclin1-GFP localization, when SK1 is coexpressed. Arrow depicts puncta in the soma. Also note beclin1-GFP puncta in neurites. Bar, 20 μm. (**b**) The puncta index was estimated by measuring the standard deviation of beclin1-GFP fluorescence intensity in a region corresponding to the neuronal soma and major neuritis in mock-, SK1-transfected neurons, and SK1-transfected neurons treated with wortmannin (20 nM). *P < 0.001 (Dunnett’s test). (**c**) The puncta index was estimated by measuring the standard deviation of mApple fluorescence intensity in a region corresponding to the neuronal soma and major neurites in mock-, SK1-transfected neurons, and SK1-transfected neurons treated with wortmannin (20 nM). N.S.—non-significant (Dunnett’s test). (**d**) Photoswitchable protein Dendra2 as a surrogate for protein turnover. Brief irradiation with short-wave-length visible light causes Dendra2 to undergo an irreversible conformational change (“photoswitch”) and emit red fluorescence that can be tracked until altered molecules are cleared. Optical pulse-chase of a primary neuron expressing Dendra2 with an automated microscope. Note the decay of red fluorescence. Bar, 50 μm. (**e**) The single-cell half-life of Dendra2-LC3 was significantly reduced by SK1 and beclin1 expression, and increased by S1PP and S1PL expression. Beclin1 was used as a positive control^11^. The change in red fluorescence intensity over time was used to calculate the half-life of Dendra2-LC3, and the half-life of Dendra2-LC3 when SK1, beclin1, S1PP or S1PL is overexpressed. *P < 0.01 (ANOVA). (**f**) Probability plot of the half-lives measurements from individual neurons. *P < 0.01 (Kolmogorov-Smirnov test).

In non-neuronal cells, overexpression of SK1 leads to enhanced autophagy^13^. We, therefore, tested if the ectopic expression of SK1 in cultured primary neurons would result in upregulated autophagy, as in non-neuronal cells. Beclin1, a constitutive protein within the pre-autophagosomal complex and a common autophagy marker^21^,^22^, fused to GFP, was expressed in three cohorts of primary neurons along with mApple, a marker of cell viability and morphology^23^. The first neuronal cohort was co-transfected with an empty plasmid; the second cohort was co-transfected with non-tagged SK1; the third cohort was co-transfected with non-tagged SK1 and treated with an inhibitor of autophagy called wortmannin. Interestingly, expression of SK1 induced the formation of beclin1-GFP-positive puncta (Fig. 1a), and wortmannin inhibited the formation of puncta (Fig. 1a,b). Notably, beclin1-GFP puncta were present in the soma as well as in neurites (Fig. 1a), suggesting that autophagosomes form in both subcellular compartments.

To score puncta formation from the fluorescence images, we measured the redistribution of beclin1-GFP into puncta by analyzing the *puncta index*^24^, defined as the standard deviation among pixels within the cellular region of interest (Fig. 1b,c). Diffuse localization corresponds to a low puncta index, whereas punctate localization indicates a high puncta index. Beclin1-GFP puncta formation was specific to beclin1 and was not an artifact of membrane blebbing, given that the diffuse distribution of mApple did not change when SK1 is expressed (Fig. 1b,c). Importantly, wortmannin inhibited SK1-associated formation of beclin1-GFP-positive puncta. These data suggest that SK1 upregulates the early stages of autophagy in primary neurons.

Since SK1 synthesizes S1P, a signaling molecule and putative enhancer of autophagy, we hypothesized that S1PP and S1PL, two S1P-metabolizing enzymes, would affect autophagy along with SK1. Therefore, we next measured if the expression of SK1, S1PP or S1PL is able to affect the basal flux through the autophagic pathway using our optical pulse-chase method (Fig. 1d)^11^,^25^. To determine if the activity of these enzymes changes autophagic flux, we transfected primary cortical neurons with i) a marker of autophagic flux Dendra2-LC3 and an empty plasmid, or ii) Dendra2-LC3 and a plasmid encoding beclin1, or iii) Dendra2-LC3 and a plasmid encoding SK1, or iv) Dendra2-LC3 and a plasmid encoding S1PP, or v) Dendra2-LC3 and a plasmid encoding S1PL. Neurons were then photoswitched, and the decay of red Dendra2 fluorescence measured. We found that neurons that overexpressed SK1 had accelerated degradation of photoswitched fluorescence, whereas overexpressed S1PP and S1PL reduced the rate of decay (Fig. 1e,f). Beclin1, used as a positive control, also slightly increased the flux, as expected^11^ (Fig. 1e,f).

### Autophagy stimulation causes the formation of SK1-positive puncta in primary neurons

**Figure 2:**
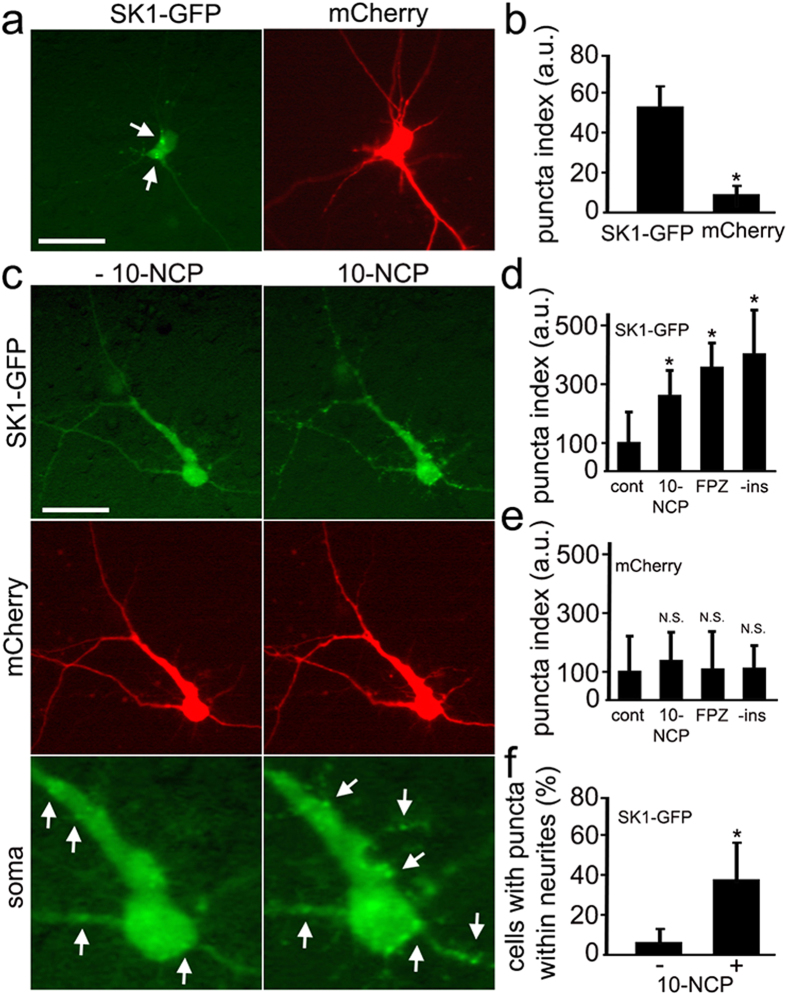
Pharmacological stimulation of autophagy causes the formation of SK1-positive puncta in primary neurons. (**a**) In a significant percentage of primary rat cortical neurons, ectopically expressed SK1-GFP exhibits a punctate appearance (left panel), whereas the distribution of a co-transfected marker of morphology mCherry (right panel) is diffuse. Bar, 50 μm. (**b**) Percentage of neurons with SK1-GFP- and mCherry-positive puncta. *P < 0.01 (t-test). (**c**) Stimulation of autophagy with 5 μM 10-NCP promotes the formation of SK1-GFP-positive puncta. Live neurons transfected with SK1-GFP and mCherry were observed before (left panel) and after (right panel) a 4-h incubation with 10-NCP. Bar, 50 μm. See bottom panel for higher magnification image of SK1 puncta. Puncta before and after the treatment are depicted with arrows. (**d**) SK1-GFP-positive puncta were efficiently formed in cortical neurons following treatment with 5 μM 10-NCP or with 5 μM fluphenazine or by incubation in insulin-free medium as reflected by the puncta index. The puncta index was estimated by measuring the standard deviation of SK1-GFP fluorescence in a region corresponding to the neuronal soma and major neurites before and after treatment with 10-NCP (5 μM, 4 h) or with fluphenazine (FPZ; 5 μM, 4 h) or after incubation in insulin-free media (-ins; 4 h). *P < 0.001 (Dunnett’s test). N.S.—non-significant (t-test). (**e**) The puncta index was estimated by measuring the standard deviation of mCherry fluorescence in the neuronal soma and major neurites. N.S.—non-significant (t-test). (**f**) Stimulation of autophagy with 10-NCP increases the number of SK1-GFP puncta not only in the soma, but also in neurites. *P < 0.001 (t-test).

To investigate how SK1 is involved in neuronal autophagy, we expressed SK1 fused to GFP in primary rat cortical neurons (Fig. 2a,b). Interestingly, SK1-GFP itself exhibited a punctate appearance in a significant percentage of neurons at steady state, whereas the distribution of mCherry, a co-transfected fluorophore, was diffuse (Fig. 2a,b). To determine whether autophagy stimulation would have any effect on the localization of SK1-GFP, we treated neurons with an autophagy enhancer, 10-NCP^26^ (Fig. 2c) and tracked the neuronal response by live imaging both before and after autophagy stimulation. Interestingly, stimulation of autophagy promoted the formation of more SK1-GFP-positive puncta (Fig. 2c). To score puncta formation from the fluorescence images, we again measured the redistribution of SK1-GFP into puncta by analyzing the puncta index before and after autophagy stimulation with 10-NCP or with fluphenazine, which is another potent inducer of neuronal autophagy^11^, or after incubation in insulin-free medium^27^, which also stimulates neuronal autophagy (Fig. 2d). Again, SK1-GFP puncta formation was specific to SK1 and was not an artifact of membrane blebbing, since the diffuse distribution of a red marker did not change in neurons stimulated with 10-NCP, fluphenazine or after incubation in insulin-free medium (Fig. 2e). The SK1-GFP puncta formed in neurons within the soma as well as in neurites (Fig. 2f). These data suggest that SK1-GFP-positive puncta represent physiologically relevant organelles and that SK1, has a functional role in the autophagic pathway.

### SK1 puncta in neurons undergoing autophagy partially colocalize with autophagosomal organelles

**Figure 3:**
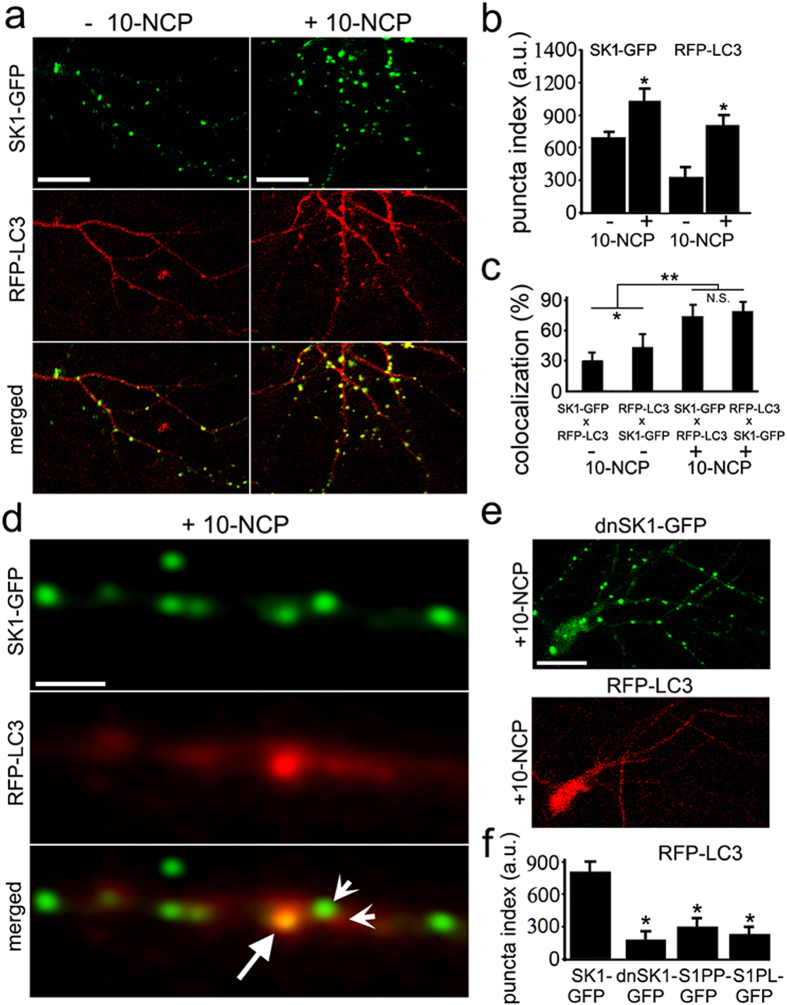
SK1 partially colocalizes with autophagosomal organelles in neurons. (**a**) Stimulation of autophagy with 5 μM 10-NCP promotes the formation of SK1-GFP-positive puncta. Neurons transfected with SK1-GFP and RFP-LC3 were treated with 10-NCP (right) or vehicle (left), fixed, and analyzed by confocal microscopy. In non-treated cells, few RFP-LC3 puncta were observed (left). In 10-NCP-treated cells, most SK1-GFP and RFP-LC3 puncta colocalized. Bar, 5 μm. (**b**) The puncta index was measured by analyzing the standard deviation of SK1-GFP or RFP-LC3 fluorescence in a region corresponding to neuronal processes. *P < 0.001 (t-test). This confirms that autophagy in neurons is upregulated. (**c**) Colocalization of SK1-GFP and RFP-LC3 signals was measured with the Coloc_2 plugin for the image processing program ImageJ/Fiji. The colocalization algorithms were run as the SK1-GFP signal against the RFP-LC3 signal (SK1-GFP x RFP-LC3) and the RFP-LC3 signal against the SK1-RFP (RFP-LC3 x SK1-GFP). *P < 0.01, **P < 0.0001 (ANOVA), N.S., non-significant. (**d**) SK1-GFP colocalizes with autophagosomal organelles in neurons. An example of a neurite in which an organelle (arrow) is both positive for SK1-GFP and RFP-LC3. Two sharp arrows depict a possible fusion event between a SK1-GFP-positive organelle and an RFP-LC3-positive organelle. Some organelles are clearly positive for SK1-GFP but not RFP-LC3 and, therefore, are not autophagosomal. Bar, 2 μm. (**e**) The dominant negative form of SK1 inhibits autophagosome formation. The expression of dnSK1-GFP prevents the formation of RFP-LC3 puncta in neurons stimulated with 10-NCP. Neurons transfected with dnSK1-GFP and RFP-LC3 were treated with 10-NCP, fixed, and analyzed with confocal microscopy. Bar, 20 μm. (**f**) The puncta index was measured by analyzing the standard deviation of RFP-LC3 fluorescence in a region corresponding to the neuronal soma and major neurites in neurons co-expressing SK1-GFP and dnSK1-GFP. The S1PP or S1PL constructs were used as controls. Neurons were stimulated with 10-NCP. *P < 0.001 (Dunnett’s test). Note that the puncta index is lower in dnSK1-GFP-expressing neurons and in neurons that express S1P-degrading enzymes.

Since neurons formed SK1-GFP puncta when autophagy was stimulated, we hypothesized that the puncta might represent autophagosomes. To test this, we co-expressed LC3 fused to TagRFP (RFP-LC3 thereafter, see methods), along with SK1-GFP in primary neurons (Fig. 3a–d). Neurons were left untreated or stimulated with 10-NCP, and fixed, and the degree of colocalization of the red and green signals was measured by confocal microscopy (Fig. 3a–d). In untreated neurons, we observed minimal colocalization of SK1-GFP puncta with RFP-LC3 puncta. In contrast, in stimulated neurons, a significant proportion of puncta was positive for both SK1-GFP and RFP-LC3, suggesting that SK1 binds to autophagosomal organelles (Fig. 3a,d). We also observed that some organelles were positive either for SK1-GFP or RFP-LC3 (Fig. 3a,d). In some rare cases, there was partial colocalization of a SK1-GFP- and RFP-LC3-positive organelle, suggesting we caught a fusion or, less likely, fission incident between the two (Fig. 3d).

Since the expression of SK1 promoted the formation of pre-autophagosomal beclin1-positive structures (Fig. 1a), we hypothesized that SK1 might be involved in the biogenesis of autophagosomes. To test this, we cloned a dominant-negative form of SK1-GFP (dnSK1-GFP; Gly81Asp) that cannot phosphorylate sphingosine^28^. Neurons were transfected with RFP-LC3 and dnSK1-GFP, treated with the autophagy enhancer 10-NCP and fixed. Interestingly, we found that neurons expressing dnSK1-GFP exhibit a punctate appearance, similar to wild-type SK1-GFP (Fig. 3e,f). In HEK 293 cells, dominant-negative SK1 is able to relocalize to the plasma membrane as well, apparently because a mutation in the catalytic center of SK1 does not affect SK1’s ability to relocalize and bind to membranes^14^. Importantly, we found no RFP-LC3-positive puncta in dnSK1-GFP-expressing neurons (Fig. 3e,f). As controls, neurons that overexpress S1PP and S1PL were used (Figs 1e–h and 3f). This finding suggests that SK1 plays a direct role at the very early stages of the autophagic pathway. Most likely, S1P generated by SK1 is necessary for the biogenesis of autophagosomal precursors.

### Autophagy stimulation leads to SK1 association with organelles positive for endosomal markers

**Figure 4:**
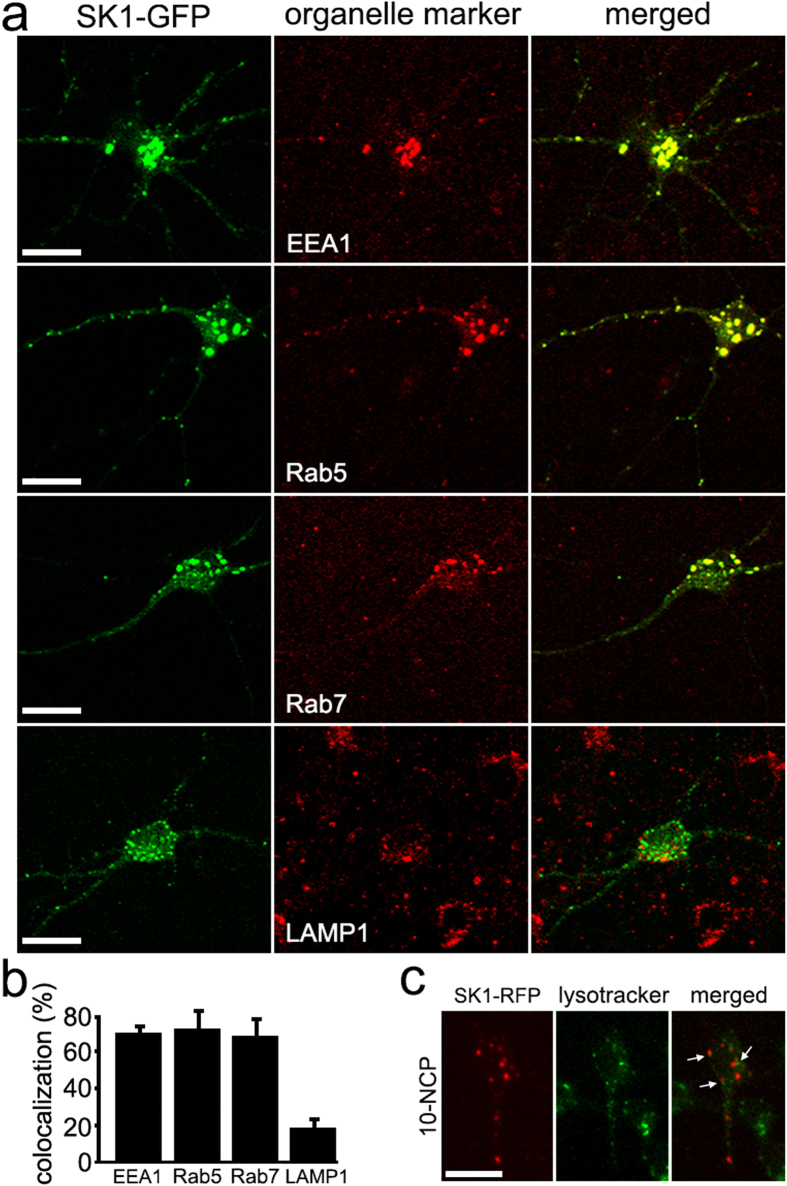
SK1 colocalizes with endosomal markers in neurons stimulated with an autophagy enhancer. (**a**) Stimulation of autophagy with 10-NCP promotes the formation of SK1-GFP-positive puncta which colocalize with early endosomes (marked by EEA1 and Rab5 staining; top panels) and with late endosomes (marked by Rab7 staining; lower middle panel). In contrast, SK1-GFP was significantly less colocalized with a marker of lysosomes (LAMP1; bottom panel). Neurons transfected with SK1-GFP were treated with 5 μM 10-NCP (4 h), fixed, stained with an antibody against EEA1, Rab5, Rab7 or LAMP1, and analyzed by confocal microscopy. Bar, 50 μm. (**b**) Colocalization of SK1-GFP and EEA1, Rab5, Rab7 or LAMP1 signals was measured with the Coloc_2 plugin for the image processing program ImageJ/Fiji. The colocalization algorithms were run as the SK1-GFP signal against an organelle marker signal (SK1-GFP x a marker). (**c**) SK1-RFP does not colocalize with the lysosomal dye Lysotracker Green DND-26. Neurons transfected with SK1-RFP were treated with 5 μM 10-NCP (2 h), then treated with 75 nM Lysotracker Green DND-26 for 30 min and imaged. Arrows indicate distinct SK1-RFP-positive and lysotracker-positive organelles, which are located nearby. Bar, 50 μm.

Our observation of discrete SK1-GFP-positive puncta, which do not colocalize with RFP-LC3 (Fig. 3a,d), suggests that SK1, in principle, can localize to cytosolic organelles distinct from autophagosomes. To investigate this further, SK1-GFP-transfected neurons were treated with 10-NCP, fixed, stained for various cytoplasmic vesicle markers and imaged by confocal microscopy. We found that 10-NCP stimulated the association of SK1-GFP with early endosomes, as labeled by the standard early endosome markers EEA1 and Rab5 (Fig. 4a,b), and with late endosomes, based on Rab7 staining (Fig. 4a,b). In contrast, SK1-GFP was significantly less colocalized with LAMP1, a marker of lysosomes (Fig. 4a,b). To make sure that we did not observe colocalization of SK1-GFP and lysosomes due to the quenching of the GFP signal in lysosomes, we cloned SK1-TagRFP construct (TagRFP is pH-insensitive; SK1-RFP thereafter). Neurons were transfected with SK1-RFP, treated with 10-NCP, and stained with a green lysotracker dye. Although SK1-RFP-positive puncta and lysotracker-positive puncta are often located nearby, these are two distinct types of organelles (Fig. 4c). These studies reveal that stimulation of autophagy also triggers the association of SK1 with early and late endosomes.

### Many endosomes make contacts with the ER

SK1 synthesizes S1P, a second messenger involved in a variety of cell signaling pathways. It was demonstrated that in non-neuronal cells S1P can mobilize calcium ions from the ER via an inositol-1,4,5-trisphosphate receptor–independent mechanism^16^,^17^, which in turn may upregulate autophagy^18^,^19^. In addition, the ER itself can provide a membrane platform for building an autophagosome by accumulating autophagosomal proteins and generating autophagosomal membranes^3^. For example, when SK1 is overexpressed, beclin1-GFP relocalized from the cytoplasm to punctate structures (Fig. 1a,b). Beclin1 is a part of a large protein complex that is assembled on ER membrane sites where an autophagosome will originate from the ER^2^,^29^. Therefore, S1P might be a signaling molecule that orchestrates the ER-dependent biogenesis of autophagosomes.

**Figure 5:**
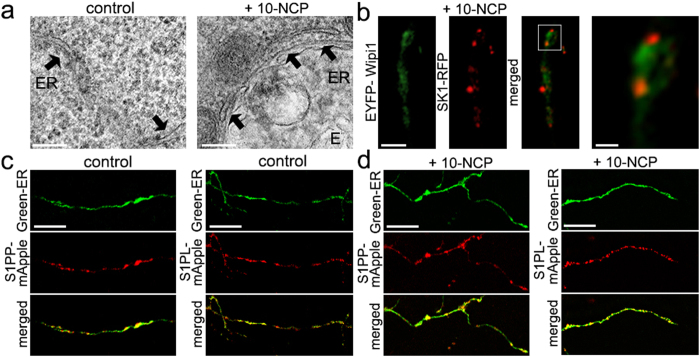
Many endosomes make contacts with the ER. (**a**) Electron micrographs of primary cortical neurons treated with a vehicle or 10-NCP (5 μM, 4 h). ER, the endoplasmic reticulum; E, endosome. Note that in an autophagy-stimulated neuron, a large endosome is surrounded by the ER. Bar, 100 nm. (**b**) In neurons stimulated with 10-NCP, SK1-RFP-positive structures make contacts with EYFP-WIPI1 structures. Neurons transfected with SK1-RFP and WIPI1-YFP were treated with 5 μM 10-NCP (4 h), fixed, and analyzed with confocal microscopy. Bar, 2 μm. Bar in the zoomed image, 0.5 μm. (**c**) Two S1P-metabolizing enzymes, S1PP and S1PL, are located on the ER. Neurons transfected with S1PP-mApple and Green-ER or with S1PL-mApple and Green-ER were fixed and analyzed by confocal microscopy. Bar, 5 μm. (**d**) S1PP and S1PL are located on the ER in 10-NCP-treated neurons. Neurons transfected with S1PP-mApple and Green-ER or with S1PL-mApple and Green-ER were treated with 10-NCP (5 μM, 2 h), fixed and analyzed by confocal microscopy. Bar, 5 μm.

It is known that endosomes communicate with the ER; these organelles make contacts and exchange signaling molecules, such as cholesterol^30^,^31^. To investigate whether stimulation of autophagy triggers the subcellular redistribution of endosomes to the ER, we treated neurons with 10-NCP or left them untreated, fixed and processed the cells for electron microscopy. Under control conditions, we found that the ER was covered with ribosomes, and no clear contacts with endosomes were observed (Fig. 5a). When autophagy was stimulated, we observed fewer ribosomes on ER membranes and more contacts between the ER and endosomes (Fig. 5a). We, therefore, conclude that the induction of autophagy triggers endosomes to make contacts with the ER. Interestingly, it was recently demonstrated that, when autophagy is enhanced, mitochondria also make contacts with the ER. At those contact sites, autophagosomes are formed^29^.

Next, we investigated if SK1-RFP-positive structures colocalize with a component of the initiation complex—WIPI1^32^,^33^. Neurons were transfected with SK1-RFP and EYFP-WIPI1, treated with an autophagy inducer 10-NCP, fixed, and analyzed by confocal microscopy. We found that many SK1-RFP-positive puncta indeed made contacts with EYFP-WIPI1-positive structures (Fig. 5b).

Our data demonstrate that SK1 enhances neuronal autophagy and suggest that SK1 may orchestrate the biogenesis of autophagosomes from the ER. We also showed that two S1P-metabolizing enzymes, S1PP and S1PL, inhibit autophagic flux in neurons (Fig. 1e–h). If autophagosomes indeed originate from the ER in neurons via SK1-dependent mechanism, we hypothesized that, similar to non-neuronal cells^15^, S1PP and S1PL are localized to the ER to ensure that unwanted S1P can be metabolized. To investigate this, neurons were transfected with S1PP-mApple and a marker of the ER, Green-ER, or S1PL-mApple and Green-ER, treated with the autophagy enhancer 10-NCP or left untreated, fixed, and analyzed by confocal microscopy (Fig. 5c,d). Expectedly, as in non-neuronal cells, both enzymes are localized to the ER in treated and non-treated neurons, suggesting that S1P can be locally metabolized to stop SK1-mediated autophagy signalling.

### SK1 and S1PL modulate the half-life of a substrate of autophagy

We and others have shown that autophagy is important for clearing misfolded protein in neurons^12^,^25^,^34^. We, therefore, hypothesized that the activity of SK1 and S1P-metabolizing enzymes may be required for the degradation of an autophagy substrate, polyglutamine-(polyQ)-expanded mutant huntingtin (mHtt), the protein that causes HD.

To test our hypothesis, we used a neuron model of HD based on the expression of an N-terminal fragment of mHtt^ex1^ fused to GFP in primary striatal neurons^23^,^25^,^26^,^35^,^36^,^37^,^38^. A similar fragment is generated in HD by aberrant splicing of mHtt mRNA^39^ and by proteolytic cleavage of mHtt protein^40^. Expression of mHtt^ex1^ produces HD-like features in mice^41^ and recapitulates many cellular HD features when expressed in neurons *in vitro*^12^.

Conventional methods are poorly suited to study the clearance of misfolded proteins because aggregation interferes with protein solubilization in the course of biochemical extractions. Cells may also die before or during an experiment, often making the measurements of the half-life misleading. To overcome these limitations, we used an optical pulse-chase technique to measure the half-life of mHtt^ex1^-Dendra2 in the presence of SK1 and S1P-metabolizing enzymes. This method has been proven to accurately measure the half-life of a misfolded protein in living single neurons^11^,^25^.

**Figure 6:**
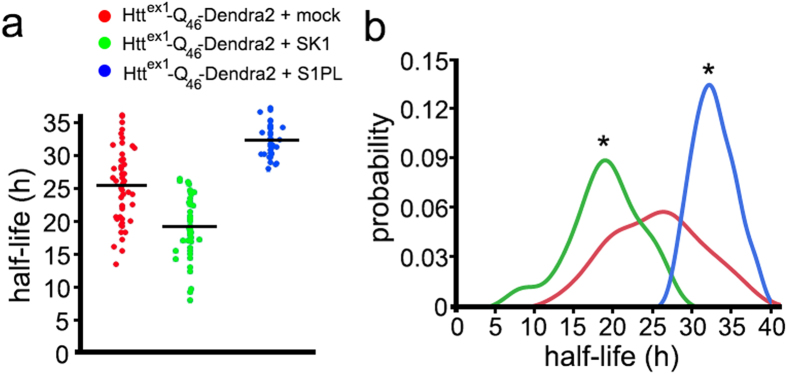
SK1 and S1PL modulate the half-life of a substrate of autophagy, Htt^ex1^-Q_46_-Dendra2. (**a**) Optical pulse labelling of striatal neurons expressing Htt^ex1^-Q_46_-Dendra2. The single-cell half-life of Htt^ex1^-Q_46_-Dendra2 was reduced by SK1 expression, and increased by S1PL expression. Neurons were transfected with either with Htt^ex1^-Q_46_-Dendra2 and an empty plasmid or Htt^ex1^-Q_46_-Dendra2 and SK1 or Htt^ex1^-Q_46_-Dendra2 and S1PL. The change in photoswitched red fluorescence intensity over time was used to calculate the half-life of Htt^ex1^-Q_46_-Dendra2 in three neuronal cohorts. *P < 0.01 (ANOVA). (**b**) Probability plot of the half-lives measurements from individual neurons. *P < 0.01 (Kolmogorov-Smirnov test).

Cultured striatal neurons were transfected with Htt^ex1^-Q_46_-Dendra2 and either an empty plasmid, SK1, the S1PP construct or S1PL. Unexpectedly, we discovered that co-expression of Htt^ex1^-Q_46_-Dendra2 and S1PP is very toxic to neurons. Perhaps, Htt^ex1^-Q_46_-Dendra2 together with sphingosine formed by S1PP, a lipid associated with apoptosis, are too insulting for neuronal health, making it impossible to measure the half-life of Htt^ex1^-Q_46_-Dendra2 under these conditions. For the other conditions, neurons were photoswitched and the decline of photoswitched Htt^ex1^-Q_46_-Dendra2 red fluorescence was measured in neurons (Fig. 6a,b). Expectedly, SK1 shortened the half-life of Htt^ex1^-Q_46_-Dendra2 and S1PL increased the half-life of Htt^ex1^-Q_46_-Dendra2. These findings indicate that the S1P pathway regulates the degradation of an autophagy substrate mHtt^ex1^ in neurons. Therefore, we conclude that the S1P pathway is a critical clearance pathway for misfolded proteins and may be a therapeutic target.

### An inhibitor of S1PL enhances survival of neurons expressing mHtt^ex1^

We previously demonstrated that an autophagy enhancer 10-NCP, the drug that upregulates SK1 (Fig. 2a), promotes neuronal survival in models of HD, spinal and bulbar muscular atrophy, and amyotrophic lateral sclerosis^11^,^25^,^42^. Here, we tested if inhibiting an S1P-metabolizing enzyme would improve neuronal survival in a neuron HD model. There are no specific inhibitors of S1PP, but there is a commercially available inhibitor of S1PL, THI. LX2931, THI’s relative compound, developed by Lexicon Pharmaceuticals, is in a phase II trial for treatment of rheumatoid arthritis patients and is well tolerated^43^.

**Figure 7:**
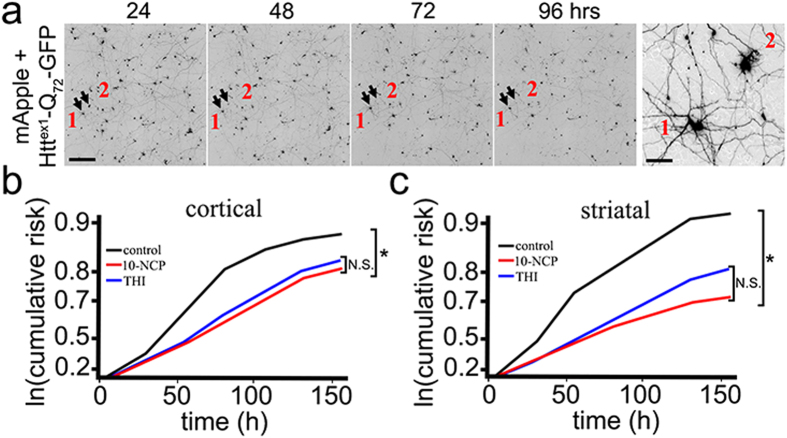
An inhibitor of S1PL, THI, enhances survival of neurons expressing Htt^ex1^-Q_72_-GFP. (**a**) An example of survival analysis. Primary cortical neurons transfected with mApple (a morphology and viability marker) and Htt^ex1^-Q_72_-GFP were tracked with an automated microscope. Images collected 24 h after transfection and 24 h thereafter demonstrate the ability to return to the same field of neurons and follow them over time. Each segmented image is a montage of non-overlapping images captured in one well of a 24-well plate. Arrows indicate randomly chosen neurons (labelled 1 and 2). These two neurons are enlarged on the right panel. Scale bar on the left panel is 400 μM. Scale bar on the left panel is 20 μm. (**b**) Cortical neurons transfected with mApple and Htt^ex1^-Q_72_-GFP were treated with 0.5 μM THI or 0.5 μM 10-NCP or vehicle. Cumulative risk for death was calculated with JMP software. 10-NCP (a positive control) and THI reduced the risk for death (i.e., improved survival) of neurons expressing mutant Htt^ex1^. *P < 0.001 (Log-Rank test). (**c**) Striatal neurons transfected with mApple and Htt^ex1^-Q_72_-GFP were treated with 0.5 μM THI or 0.5 μM 10-NCP or vehicle. Cumulative risk for death was calculated with JMP software. 10-NCP and THI reduced the risk for death of neurons expressing mutant Htt^ex1^. *P < 0.001 (Log-Rank test).

Striatal and cortical neurons were transfected with mApple (a morphology and viability marker) and Htt^ex1^-Q_72_-GFP (Fig. 7a). Neurons were then treated with vehicle, 0.5 μM 10-NCP (a positive control), or 0.5 μM THI, and imaged every 24 h with an automated microscope. Cumulative hazard plots revealed that neurons expressing Htt^ex1^-Q_72_-GFP that were treated with 10-NCP or THI survived better than control neurons (Fig. 7b,c). These findings indicate that modulating the S1P pathway might have therapeutic benefit in HD.

## Discussion

Here, we identified a novel pathway for the regulation of neuronal autophagy. Overexpression of SK1, a kinase that generates S1P, enhances the formation of pre-autophagosomal beclin1-positive structures and increases flux through the autophagic pathway, and S1PP and S1PL, enzymes that degrade S1P, reduce autophagic flux. In contrast, expression of a dominant-negative form of SK1 inhibits autophagosome formation, suggesting that SK1 plays a role in the biogenesis of autophagosomes. A small-molecule autophagy enhancer triggers relocalization of SK1 to organelles positive for endosomal or autophagosomal markers or both, suggesting that endosomes and autophagosomes fuse in neurons undergoing autophagy. When autophagy is upregulated, endosomes make contacts with the ER, suggesting that endosomes might provide the ER with S1P for the biogenesis of autophagosomes. Remarkably, both S1PP and S1PL are located on the ER, which ensures that unwanted S1P can be locally metabolized to stop SK1-mediated autophagic signaling. Finally, we demonstrate the importance of the S1P pathway in the autophagic degradation of an autophagy substrate, the mHtt fragment Htt^ex1^. Our results suggest that the S1P pathway may be a target for therapy development in HD and in neurodegenerative disorders in general.

There are two sphingosine kinases in mammalian cells, SK1 and SK2. Double knockout of these kinases is embryonically lethal, which illustrates the importance of these kinases in cellular biology. In contrast to SK2, which appears to be mainly present in the nucleus and mitochondria, SK1 is localized to the cytosol. SK1 can be activated by a variety of factors, including growth factors, cytokines, and numerous GPCR ligands^15^. ERK1/2 kinase directly phosphorylates SK1^44^, which results in increased SK1 enzymatic activity and in translocation of SK1 to the plasma membrane in non-neuronal cells. Interestingly, ERK1/2 has been implicated in HD. ERK1/2 is hyperphosphorylated in HD cells and pharmacological activation of ERK1/2 is neuroprotective, suggesting that cells cope with mHtt by increasing the activity of ERK1/2 cascade^45^,^46^. Our results are consistent with the concept that the protective properties of ERK1/2 may be also governed by the pro-autophagic functions of SK1. Future studies in neurons will address this possibility.

**Figure 8:**
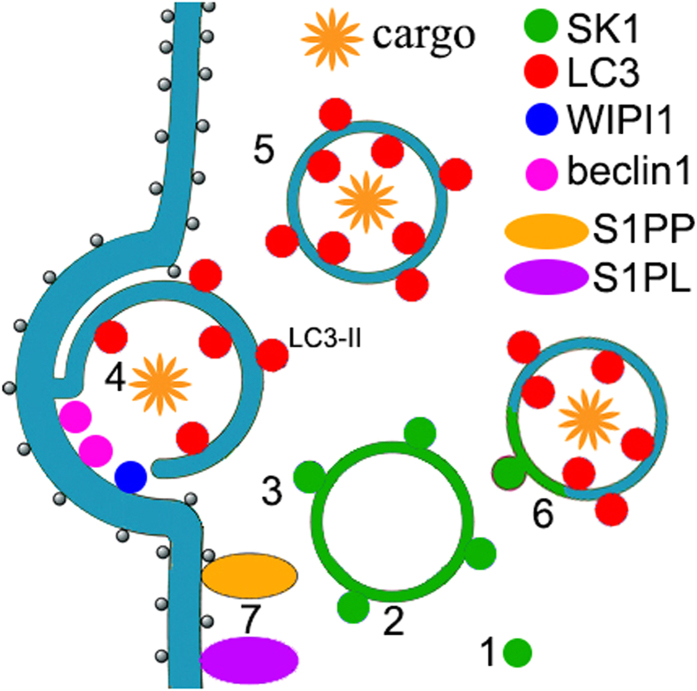
A possible mechanism of SK1-mediated autophagy. Pharmacological stimulation of autophagy results in activation of SK1 (1), which then relocalizes to endosomes (2). An endosome then may make contacts with the ER surface, where WIPI1 and beclin1 protein complexes are assembled (3), resulting in the biogenesis of an pre-autophagosomal structure (4). Once formed (5), a LC3-positive autophagosome (5) may fuse with the endosome to form SK1- and LC3-positive amphisomes (6). S1PP and S1PL are localized to the ER, ensuring that unwanted S1P can be locally metabolized to stop S1P signaling (7).

The origin of the autophagosomal membrane and the biogenesis of an autophagosome are topics of enormous interest in the autophagy field. Our data suggest a possible mechanism of SK1 in neuronal autophagy (Fig. 8). Pharmacological stimulation of autophagy results in the activation of SK1 and subsequent translocation of SK1 to the membranes of endosomes. The intracellular receptors for S1P, which are ligand-gated calcium channels, are located on the ER surface^17^. An endosome then may make contact with the ER surface, perhaps releasing calcium that is necessary for the formation of an autophagosome. In addition, at a contact site, WIPI1 and beclin1 protein complexes are assembled, resulting in the biogenesis of an autophagosome. Interestingly, two S1P-metabolizing enzymes, S1PP and S1PL, are also located on the ER^15^—which ensures that unwanted S1P can be locally metabolized to stop S1P signaling. Once formed, some autophagosomes then may fuse with endosomes, resulting in the formation of SK1- and LC3-positive amphisomes. A similar ER-dependent mechanism of autophagosome biogenesis has been shown before^3^, but our findings demonstrating the involvement of SK1 and endosomes make the picture more complete.

There has been an immense interest in autophagy in the recent years. Besides being a critical fundamental pathway for homeostasis in healthy cells, dysregulated autophagy is involved in many human diseases, including neurodegenerative disorders, cancer, infections, and disorders of metabolism. In the field of neurodegeneration, research has focused on how to enhance autophagy in neurons and, therefore, promote clearance of toxic misfolded proteins that cause neurotoxicity. In our study, we identified a new protein, SK1, which regulates neuronal autophagy and promotes the degradation of an autophagy substrate that causes HD. These findings implicate SK1 as a potentially promising target for therapeutic development in HD and other neurodegenerative disorders in which autophagic processes are disrupted.

## Materials and Methods

### Plasmids and Chemicals

10-NCP (10-(4′-(N-diethylamino)butyl)-2-chlorophenoxazine) was from Calbiochem. Fluphenazine was from Sigma. Wortmannin was from Selleck Chemicals. THI (2-Acetyl-5-tetrahydroxybutyl-imidazole) was from Cayman Chemical. Antibodies against EEA1 were from Cell Signaling (#2411; 1:100), against Rab5 were from Cell Signaling (#2143; 1:100), against Rab7 were from Cell Signaling (#9367; 1:100), against LAMP1 were from Cell Signaling (#9091; 1:100). Secondary antibodies were from Invitrogen; Alexa Fluor 555 (#A21422; 1:1000); Alexa 488 donkey (#A21206; 1:1000). LysoTracker Green was from Life Technologies (#L-7526). pEGFP-SK1, a gift from Dr. Sarah Spiegel (Virginia Commonwealth University), was subcloned into the pGW1 vector. pGW1-SK1-TagRFP (described as SK1-RFP in the text and in the figure legends) was cloned from pTagRFP-N (Evrogen) and pGW1-SK1-GFP. pGW1-beclin1-GFP was cloned from pEGFP-beclin1 (a gift from Dr. Zhenyu Yue, Mt. Sinai Medical School). The dominant negative form of SK1 (GFP-tagged) was cloned by mutating Gly81 to Asp^28^. pGW1-SK1 was cloned from pGW1-SK1-GFP by removing the GFP tag. pGW1-TagRFP-LC3 (described as RFP-LC3 in the text and in the figure legends) was cloned from pTagRFP-C (Evrogen) and from pEGFP-LC3 (a gift from Dr. Tamotsu Yoshimori, Osaka University), which was described^47^. The TagRFP, unlike many other red fluorescent proteins, does not aggregate^48^, which makes it ideal for tagging the LC3 protein. pGW1-mCherry was described^12^. pmApple was from Dr. Kurt Thorn (University of California, San Francisco). pcDNA3-S1PL-GFP, a gift from Dr. Julie Saba (Children’s Hospital Oakland Research Institute), was subcloned into the pGW1-mApple or pGW1-GFP vector. pcDNA3-S1PP, a gift from Dr. Lina Obeid (Stony Brook University), was subcloned into the pGW1-mApple vector or pGW1-GFP vector. pGW1-Htt^ex1^-Q_72_-GFP and pGW1-Htt^ex1^-Q_46_-Dendra2 were described^12^,^25^. pNeonGreen-ER (Green-ER in the text) was from Allele Biotechnology. pGW1-Dendra2 and pGW1-Dendra2-LC3 were described^25^. pCMV-EYFP-WIPI1 was cloned from pMXs-IP-GFP-WIPI1, which was obtained from Addgene (#38272).

### Neuronal Cultures and Transfection

Cortices from P0 rat pups and striata from rat embryos (E17–18) were dissected, dissociated, and plated on 24-well tissue-culture plates (7 × 10^5^/well) coated with poly-D-lysine (BD Biosciences, San Jose, CA) as described^12^. Neurons were grown in modified neuronal growth medium made from Neurobasal Medium (Life Technologies), B-27 supplement (Life Technologies), GlutaMAX (Life Technologies), and penicillin-streptomycin (Life Technologies). Primary cultures were transfected after 4 days with Lipofectamine (Invitrogen) and with a total of 1–2 μg of plasmid DNA per well, as described^12^,^23^,^25^. Some cultures were treated with 10-NCP (5 μM, 4 h), with fluphenazine (5 μM, 4 h) or incubated in insulin-free medium (B-27 supplement, minus insulin, 4 h). Some cultures were treated with wortmannin^49^,^50^ (20 nM) 8 h after transfection and analysed 24 hours after transfection.

### Immunocytochemistry

Cultured neurons were fixed with 4% paraformaldehyde for 15 min at room temperature, then incubated in the quenching buffer (PBS, 100 mM glycine) for 10 min, permeabilized in PBS containing 0.1% Triton X-100, and blocked for 1 h in PBS containing 10% serum from the host species of a secondary antibody. Cells were incubated with a primary antibody diluted in blocking buffer at 4 °C overnight. Cells were then washed with blocking buffer, incubated with a secondary antibody in blocking buffer for 1 h at room temperature, washed again 3 times for 5 min. The coverslips were mounted with an anti-fade mounting medium (Life Technologies) and slides were analyzed.

### Colocalization with Lysosomes

Since there is a possibility that SK1-GFP does not colocalize with lysosomes due to quenching of the GFP signal, the S1K-TagRFP was cloned to test if SK1-TagRFP colocalizes with green lysotracker. Cortical neurons were transfected with pGW1-S1K-TagRFP and treated with 5 μM 10-NCP for 2 h. Neurons were then treated with 75 nM Lysotracker Green DND-26 (Life Technologies, #L-7526) for 30 min and imaged.

### Fluorescence Microscopy

Live cell imaging was performed with a Nikon TE2000E-PFS microscope (a long-working-distance Nikon CFI S Plan Fluor 20 × (NA 0.45) objective, a 300 W Xenon Lambda LS illuminator (Sutter Instruments, Novato, CA)) and the EVOS microscopy system (Life Technologies). Fixed cells were analyzed with a Zeiss LSM510 confocal microscope. Photoswitching of Dendra2 and Dendra2-LC3 was performed as described^25^. Studying autophagy in neurons is technically difficult. Our inability to biochemically measure the levels of LC3-II in transfected neuronal cultures led us to develop the optical-pulse-chase method based on a photoswitchable marker. Importantly, conventional assays are often very toxic for primary neurons and, therefore, insensitive. The analysis of the half-life of Dendra2-LC3 (e.g., autophagic flux) avoids the limitations such as neurotoxicity associated with drug treatments, thereby making it possible to study autophagy in neurons.

### Image Analysis

Puncta formation and puncta indexes were analyzed as described^12^. Briefly, the redistribution of SK1-GFP into punctate structures was reflected by the puncta index, which is the standard deviation of the intensities measured among pixels within the cellular region of interest before and after treatment. Diffuse localization corresponds to a low puncta index, and punctate localization corresponds to a high puncta index. In a standard experiment, 10–40 neurons from three experiments were analyzed^11^,^25^.

The protein levels were measured with ImageJ, an open-source image-processing program.

The decays of photoswitched and non-photoswitched Dendra2 fluorescence were measured by measuring “red” fluorescence intensity over a region of interest corresponding to the neuronal soma and major neurites (fluorescence of non-photoswitched “green” molecules served as a guide for drawing the region of interest). The intensities were plotted against time and transformed into log values^11^,^25^. At least 30 neurons from three experiments were analyzed.

Colocalization of SK1-GFP and the endosomal markers, SK1-GFP and LAMP1, SK1-GFP and RFP-LC3, SK1-GFP and RFP-LC3 was measured with the Coloc_2 plugin for ImageJ/Fiji.

To determine whether SK1 and S1P-metabolizing enzymes regulate degradation of mHtt^ex1^-Dendra2, striatal neurons were cultured as described above, transfected either with pGW1-mHtt^ex1^-Q_46_-Dendra2 and with an empty plasmid or with pGW1-mHtt^ex1^-Q_46_-Dendra2 with a plasmid encoding SK1, S1PP or S1PL. Neurons were photoswitched and analyzed with automated microscopy 36 h thereafter, as described above^25^,^51^.

### Electron Microscopy

Cortical neurons were grown as described above. Neurons were treated with 5 μM 10-NCP for 4 h. Cells were then washed with PBS and fixed with a solution containing 3% glutaraldehyde and 2% paraformaldehyde in 0.1 M cacodylate buffer. Cells then were washed in 0.1 M cacodylate buffer and treated with 0.1% Millipore-filtered buffered tannic acid, postfixed with 1% buffered osmium tetroxide for 30 min, and stained with 1% Millipore-filtered uranyl acetate. The samples were washed several times in water, then dehydrated in increasing concentrations of ethanol, infiltrated, and embedded in LX-112 embedding medium. The samples were polymerized in a 60 °C oven for 2 days. Ultrathin sections were cut in a Leica Ultracut microtome (Leica, Deerfield, IL), stained with uranyl acetate and lead citrate in a Leica EM Stainer, and examined in a JEM 1010 transmission electron microscope (JEOL, USA, Peabody, MA) at an accelerating voltage of 80 kV. Digital images were obtained using AMT Imaging System (Advanced Microscopy Techniques Corp, Danvers, MA).

### Survival Analysis

Striatal and cortical neurons were transfected with mApple (a morphology and viability marker) and Htt^ex1^-Q_72_-GFP. Neurons were treated with vehicle (DMSO) or with 0.5 μM 10-NCP or with 0.5 μM THI and imaged every 24 h for 1 week. For tracking the same neurons over time, an image of the fiduciary field with neurons on the plate was collected at the first time-point and used as a reference image. Each time the same plate was imaged thereafter, the fiduciary image was aligned with the reference image. Neurons that died during the imaging interval were assigned a survival time (the period between transfection and their disappearance from an image). These event times were used to obtain the exponential cumulative survival graphs and analyzed for statistical significance by Log-Rank test. Statistical analysis used JMP (SAS Institute)^12^,^23^,^25^. Curves were generated in JMP. Experiments were repeated 4 times with more than one hundred neurons analyzed per an experiment.

### Ethics Statement

Rats were maintained in accordance with guidelines and regulations of the University of Texas, Houston (the protocol number #AWC-13–122). All experimental protocols were approved by the University of Texas, Houston. The methods were carried out in accordance with the approved guidelines.

## Additional Information

**How to cite this article**: Moruno Manchon, J. F. *et al.* Cytoplasmic sphingosine-1-phosphate pathway modulates neuronal autophagy. *Sci. Rep.* **5**, 15213; doi: 10.1038/srep15213 (2015).
